# Bracing and non-surgical management of scoliosis in Canada: Early detection, access inequities, and the need for interdisciplinary reform

**DOI:** 10.33137/cpoj.v8i2.46590

**Published:** 2025-12-31

**Authors:** A Lebel, M Kline, J Boucher, J Carberry, N Adulovic, JA Dermott

**Affiliations:** 1 Scoliosis Physiotherapy & Posture Centre, Ottawa, ON, Canada.; 2 Independent Researcher, Barrie, ON, Canada.; 3 Centre intégré de santé et de services sociaux de l'Abitibi-Témiscamingue, Rouyn-Noranda, Qc, Canada.; 4 Faculty of Medicine, Palacký University, Czech Republic.; 5 Faculty of Science, University of Ottawa, Ottawa, ON, Canada.; 6 The Hospital for Sick Children, Toronto, ON, Canada.

**Keywords:** Scoliosis, Spinal Orthosis, Physiotherapy, Adolescent Idiopathic Scoliosis, Bracing, Orthotics, Physiotherapy Scoliosis-Specific Exercise, PSSE, Healthcare Access, Health Policy, Spinal Fusion, Canada

## Abstract

Bracing remains the cornerstone of non-surgical management for adolescent idiopathic scoliosis (AIS) with an aim to minimize the risk of progression and to avoid spine surgery. In Canada however, one third to half of patients present with curve magnitudes too severe for optimal brace treatment, resulting in higher than necessary surgical volumes. High-cost spine surgeries are fully funded while non-surgical management is not. This Professional Opinion article highlights systemic barriers to early detection that limit opportunity for non-surgical management in Canada and ultimately drive up healthcare spending. In Canada, there is an urgent need for a coordinated national strategy to re-establish routine scoliosis screening, ensure equitable public funding for treatment and expand professional training in non-surgical spinal care.

## INTRODUCTION

Adolescent idiopathic scoliosis (AIS) is a three-dimensional spinal deformity defined by a Cobb angle >10° with vertebral rotation, in youth at least 10 years of age.^1^ During growth, there is a high risk of curve progression, that if severe, may necessitate surgery. While its cause is unknown, genetic, neuromuscular, and biomechanical factors contribute. AIS affects approximately 2–3% of children and adolescents globally.^2^

Most cases are initially asymptomatic and often detected through visible asymmetries such as uneven shoulders, rib prominence, or trunk imbalance. Scoliosis Research Society (SRS) guidelines suggest that curves <20° (Risser 0–1) are observed, 20–40° (Risser 0–3) are managed with bracing, and curves ≥50° are considered surgical candidates.^3^ Bracing refers to a custom, three-dimensional, rigid thoracolumbosacral orthosis (TLSO). The landmark Bracing in Adolescent Idiopathic Scoliosis Trial (BrAIST) publication^4^ demonstrated that 72% of braced patients met treatment success (i.e., did not progress to surgical range), compared to 48% in the observation group. Additional studies have shown that success rates can exceed 80% when patients adhere to brace wear of at least 18 hours per day.^5^ In the context of AIS, the gold-standard surgical intervention is posterior spinal fusion (PSF). Typically, this involves dual solid rods held by segmental pedicle screws with autograft bone used for fusion of the instrumented spine. SOSORT 2016 guidelines emphasize that early identification of mild to moderate scoliosis and individualized non-surgical management are essential to prevent curve progression and reduce the need for surgery.^6^ The inclusion of physiotherapeutic scoliosis-specific exercises (PSSE) should be considered as an adjunct to bracing^7^ and has been shown to improve postural control, reduce pain, and enhance brace effectiveness.^8,9^

More than thirty percent of Canadian patients are diagnosed late, with curves too severe for effective non-surgical treatment^10,11^ an issue that was further exacerbated by the COVID-19 pandemic.^12^ This illustrates the importance of prioritizing early detection and non-surgical management before surgical intervention. This paper highlights systemic barriers to early AIS identification and compares associated costs and funding models.

## UNIVERSAL HEALTHCARE IN CANADA

Canada has a publicly funded universal healthcare system that provides residents with access to medically necessary hospital and physician services, free at the point of care.^13^ The federal government sets national principles under the Canada Health Act, but care delivery and administration are the responsibility of the provinces and territories, resulting in 13 distinct health systems. Funds are generated through both federal- and provincial-level taxes, and administration operates on a non-profit basis by public authorities. In addition to public coverage, some individuals hold private insurance or extended health benefit plans, often obtained through employers, which may cover services not included under the publicly funded system.

## ACCESS INEQUITIES AND EARLY DETECTION PRACTICES

### Scoliosis Screening

The Canadian Task Force on Preventative Health Care (CTFPHC) notes insufficient evidence to recommend for or against scoliosis screening; its statement has been archived and not updated since 1994.^14^ Studies indicate that non-medical observers often first notice asymmetry, but when primary care providers (PCPs) screen for AIS, they detect smaller curves with greater accuracy.^10,15^ However, PCP screening in Canada is inconsistent, partly due to the Greig Health Record recommending against screening in asymptomatic youth, arguing that mild scoliosis is typically benign and bracing/exercise does not improve pain or quality of life.^16^ This is problematic because AIS is asymptomatic, many patients still require surgery, and bracing primarily aims to prevent curve progression. In contrast, reviews by the SRS, American Academy of Orthopaedic Surgeons (AAOS), American Academy of Pediatrics (AAP), and Pediatric Orthopaedic Society of North America (POSNA), and recommendations from the SRS International Task Force, support screening as technically and clinically valuable.^17,18^ Although the U.S. Preventative Services Task Force (USPSTF) notes ‘uncertainty about the balance of benefits and harms’ of screening,^19^ the preventative care guidelines used by American pediatricians do include routine back examinations to screen for scoliosis for 11–18-year-olds.^20^

School-based screening in Canada was largely discontinued during the 1980s, initially intended to detect curves early in children aged 10–13 years.^21^ At the time, programs were considered costly, required trained personnel, and raised concerns about variable accuracy, high false-positive rates, and unnecessary radiation from follow-up x-rays.^22,23^ Insufficient evidence at the time that early detection improved outcomes further supported discontinuation.

Technological and research advances have since addressed many of these concerns. Low-dose EOS imaging^24^ reduces cumulative radiation exposure. AI-based screening apps and virtual tools are being developed and may augment the need for frequent X-rays for monitoring. Missed opportunities for early detection have contributed to longer wait times, more invasive treatments, and increased healthcare costs^25,26^ underscoring the value of timely screening.

The CTFPHC has recently undergone a review to enhance Canada's preventative health framework, and the External Expert Review recommended modernization of archived guidelines.^27^ The newly formed Canadian Scoliosis Screening Coalition, endorsed by the Canadian Paediatric Spine Society, aims to promote early detection and prevent harmful delays in access to care (https://scoliosiscanada.ca/).

Internationally, organizations including SRS, AAOS, AAP, and POSNA recommend screening girls twice at ages 10 and 12, and boys once at 13 or 14.^17,28^ Screening is standard or optional in countries such as Singapore, Belgium, Croatia, Cyprus, Malta, Slovenia, Sweden, Greece, Italy, the Netherlands, Spain, and Turkey.^22^ A meta-analysis of 34 studies found that scoliosis detected during adolescent screening reduced the likelihood of requiring spinal fusion by 73%.^29^

### Provincial Inconsistencies in Funding and Prescribing Non-Surgical Management

Each provincial-level healthcare system determines its own coverage policies for orthotic devices. While all surgical costs associated with spinal fusion are publicly funded, custom scoliosis braces are not always eligible for reimbursement, leaving families to pay $3,000–$6,000 CAD or more out-of-pocket. This disparity puts families at risk of not being able to afford non-surgical care, even though it may prevent the need for surgery, which ultimately is more costly at a system level.^30^

Government funding for scoliosis bracing is highly variable across Canada and is contingent on prescriptions from recognized professionals, generally a physician or nurse practitioner, fabricated by an approved provider. Quebec has the most comprehensive public funding, at two braces per growth period^31^ supporting day–night bracing strategies. In Manitoba the cost of a scoliosis brace is covered under the Manitoba Health Prosthetic and Orthotic Program.^32^ Ontario residents are eligible for 75% coverage through the Assistive Devices Program (ADP)^33^ with a new brace allowed to accommodate growth, approximately once a year. Alberta employs a structured, cost-share system through the Alberta Aids to Daily Living (AADL) program, providing two braces every two years for pediatric patients with a modest family contribution that is tiered by family taxable income.^34^ British Columbia, the Atlantic provinces and the territories offer no standardized provincial funding, leaving families reliant on private insurance or out-of-pocket payment. In most provinces, families that have qualified for social assistance, do have the cost of the brace covered. Indigenous children, regardless of geographic location can access Non-Insured Health Benefits for full coverage of the cost of orthotic devices.^35^

Despite criteria to guide non-surgical treatment decisions,^7^ brace prescription practices in Canada remain highly variable and appear to be influenced as much by local clinical practice patterns as by curve characteristics.^36^ From the author's experience, spine surgeons differ considerably in the lower threshold to start bracing, whether bracing is offered for larger curves, and in which brace designs are considered effective. For example, a patient with a fifty-six–degree adolescent idiopathic scoliosis curve was referred for bracing in one city, while a comparable curve in another was deemed “brace-ineffective” and managed with surgery as the only option; in a third location, bracing was considered possible but surgical intervention was anticipated. When reassessed eight months later in a different city, the same patient was advised that surgery might not be required, as bracing combined with PSSE had been effective to date. There is no uniformity to the prescription of bracing and PSSE across the country. These discrepancies highlight the absence of national standardization and illustrate how geographically dependent clinical philosophies can substantially influence prioritization of non-surgical scoliosis care.

There is a need for national guidelines and equitable funding frameworks to ensure that youth with scoliosis receive timely and appropriate orthotic management regardless of geographic location.

### Workforce Shortages and Training Gaps

Canada faces a significant shortage of healthcare professionals trained in non-surgical scoliosis management.^37^ Few orthotists receive comprehensive training in scoliosis-specific bracing despite the long learning curve required for high in-brace correction. Achieving in-brace correction of 30–50% is predictive of long-term success.^38,39^ More recent evidence extends these findings, with studies of high-correction three-dimensional bracing, for example, a Cheneau-style braces, reporting average in-brace corrections of 60-70% and long-term success rates exceeding 90% using SRS criteria, outperforming standard thoracolumbosacral orthoses and challenging traditional bracing thresholds.^40,41^ These data highlight the critical role of orthosis provision, underlying biomechanical principles, and orthotist expertise in determining outcomes, variables that are not consistently available across Canadian provinces.^36^ Furthermore, brace designs range from traditional Boston braces to newer three-dimensional systems such as Chêneau and Rigo-Chêneau, Gensingen and Gomez designs, and night-time braces such as the Providence brace. Despite their established use and strong supporting evidence in the international literature, adoption of these contemporary designs remains limited in Canada, and no national data on how brace design decisions are made across the country.

Only a small proportion of physiotherapists are certified in evidence-based PSSE methods such as Schroth, SEAS, or Lyon.^42^ Nurse practitioners and other non-surgical providers also remain underutilized in early detection and referral pathways. Théroux et al. found that 40% of healthcare providers mainly family physicians and physiotherapists did not feel confident managing adolescents with idiopathic scoliosis.^43^ Diagnostic quality creates further challenges: community X-ray reports identify brace-range scoliosis accurately only 65.8% of the time, with nearly one third of candidates missed due to curve underestimation. Inaccuracies in Cobb angle readings increase the odds of late AIS presentation 3.5-fold,^44^ reducing opportunities to stabilize curves before surgical thresholds are reached.

Improving access requires better education for primary-care providers (PCP) on when to assess, monitor, and refer patients to spine specialists. This starts with updating screening recommendations within the Greig Health Record, the clinical tool used by Canadian PCPs to guide preventative health examinations of youth 10–14 years of age. Routine screening combined with family guidance on simple at-home checks would further support earlier detection.

Technology-assisted tools, including smartphone posture apps and AI-supported screening platforms, should be validated as they may help reduce diagnostic delays. Emerging AI systems may also improve radiological accuracy; Wu et al. demonstrated that their two-step Augmented U-Net model measured over 90% of 3-foot standing spine X-rays within 10° and over 72% within 5° of benchmark values.^45^ Establishing clear interdisciplinary referral pathways is essential to ensure timely non-surgical management. Given that most scoliosis does not require surgery, expanding task-shifting strategies would enable appropriately trained non-physician specialists to manage suitable cases, improving access while maintaining care quality.

### Early Detection and Referral Pathway for Non-Surgical Scoliosis Care Surgical Costs

In contrast to the funding of non-surgical treatment, PSF is fully covered by public health insurance across Canada. The Canadian Institute for Health Information (CIHI) estimates average surgical costs between $60,000–$120,000 CAD per case, excluding long-term rehabilitation,^25^ revision procedures and complications (infections, hardware failure).^46^

A current challenge in scoliosis healthcare is the extensive wait time for surgery, with many patients waiting over two years. These prolonged delays increase the risk of complications and are estimated to cost the healthcare system $44.6 million CAD, according to a 2023 report by The Conference Board of Canada.^26^ Complication rates range from 6–25% in adolescents to over 39% in adults with spinal deformity.^47,48^ Reoperation rates in this five-year cohort of patients were 9.9%. The most common indications for reoperation were infection at 4.5% (2.4% delayed infections and 2.1% acute infections).^49,50^ Infection-related readmissions may cost an additional $75,172 CAD per episode.^51^ Nadler et al. found that at a single centre in Ontario, for each late presenting patient (curve magnitude 50°) that is instead seen as an ideal brace candidate, over $32 000 CAD is saved.^30^

In Canada, at least one-third of patients are first diagnosed with scoliosis only after their condition has progressed to a severe curvature, a stage at which surgical intervention is often the most appropriate or only viable treatment option. This late presentation reflects systemic gaps in early detection and preventive care. Unlike several other countries, Japan, Hong Kong, Singapore, and several European countries including Sweden, Greece, Italy, Spain, Turkey, and in 24 States in the US,^52^ Canada does not have a consistent, nationwide scoliosis screening program, resulting in missed opportunities to identify and manage the condition during its early, more treatable stages. In addition, access to publicly funded non-surgical interventions, such as bracing and scoliosis-specific physiotherapy, is limited or inconsistent across provinces. These gaps are compounded by insufficient training and availability of healthcare practitioners who specialize in evidence-based, non-surgical scoliosis care.

Despite these shortcomings, high-cost surgical interventions, including spinal fusion procedures, are fully covered under Canada's publicly funded healthcare system. This imbalance in funding priorities places a greater emphasis on reactive, invasive treatment rather than proactive, conservative management. Review of provincial funding practices for orthotics further demonstrated that even when scoliosis is detected early, significant interprovincial disparities persist. Variations in coverage, referral pathways, and availability of specialized services continue to restrict equitable access to non-surgical management options across the country, ultimately influencing patient outcomes and reinforcing regional inequities within the Canadian healthcare system.

Canada's current healthcare framework inadvertently favours surgical intervention by not regulating regular, routine healthcare screening, and by fully covering fusion surgery while leaving bracing and physiotherapy largely unfunded. The discontinuation of school-based screening programs, combined with the recommendation against scoliosis screening for routine preventative health care assessments in the Greig Health Record, presents a substantial barrier to ensuring that children receive the preventive care they require. This imbalance undermines early detection and non-surgical management that could prevent curve progression to surgical range, in most moderate cases. In the absence of systematic screening, Canada faces increasing financial strain due to the high costs associated with scoliosis surgeries and their related complications. Moreover, Canadian patients and their families are subjected to invasive treatments that might otherwise be avoided through timely detection, reflecting a significant gap in the nation's approach to pediatric preventive healthcare.

With the significant advancements in scoliosis research and technology since the 1980s, it is imperative that current practices evolve accordingly. We now possess the knowledge and tools necessary to better support these children, and healthcare policy must reflect the information available to us.

Evidence from multiple jurisdictions demonstrates that scoliosis screening programs are associated with higher rates of non-surgical management and lower rates of surgical intervention.^29^ In Norway, the proportion of patients undergoing surgery increased substantially during periods without screening compared with periods when screening was in place, with reported surgical rates rising from approximately one third to nearly two thirds of patients.^53^

Similarly, population-based data from Hong Kong indicate that only about fifteen percent of patients identified through screening ultimately required surgery,^54^ compared with roughly sixty percent of those diagnosed outside a screening context in Norway.^55^ Importantly, earlier detection through screening facilitates timely initiation of conservative management. Bracing has been shown to reduce progression of curves to the surgical threshold, with randomized controlled trial data demonstrating success rates exceeding seventy percent overall and approaching ninety percent among highly compliant patients.^4,56^

Comparable long-term outcomes have been observed in large cohort studies, including Norwegian population-based data. Together, this evidence supports early detection through screening as a means of enabling timely bracing, preserving spinal mobility, avoiding surgical complications, and potentially reducing healthcare costs. Health economic analyses further suggest that selective screening strategies targeting higher-risk populations, such as adolescent girls, may offer an efficient and cost-effective approach to reducing the burden of surgical treatment in adolescent idiopathic scoliosis.^55^

**A National Scoliosis Strategy Should Include:**

- Reintroduction of scoliosis screening to facilitate early detection.
- Public funding for custom scoliosis bracing and PSSE to ensure access to effective non-surgical treatment regardless of socio-economic status and location in Canada.
- Expanded interdisciplinary education and training for healthcare professionals including orthotists, physiotherapists, family physicians, and nurse practitioners to improve care delivery.
- National data collection on outcomes and costs to guide evidence-informed policy and ensure equitable access across regions.

Such reforms would prioritize non-surgical AIS management with an aim to reduce the number of children progressing to surgical thresholds.

International comparisons underscore the importance of system-wide infrastructure and robust public funding in the effective management of adolescent idiopathic scoliosis (AIS). In Germany, high-correction bracing and physiotherapeutic scoliosis-specific exercise programs are publicly funded^57^ and widely accessible, supporting early, conservative intervention. Similarly, in England, scoliosis bracing is fully covered through the National Health Service, ensuring that eligible adolescents can access prescribed orthotic treatment without direct financial burden.^58^ In contrast, Quebec is currently the only jurisdiction in Canada that offers comparable public coverage for scoliosis bracing.

To meaningfully prioritize non-surgical AIS management, Canada faces a two-fold challenge: improving early detection of curves amenable to conservative treatment and establishing equitable, publicly funded access to evidence-based non-surgical interventions. Addressing both detection and funding gaps is essential to reducing progression to severe deformity and reliance on costly surgical care.

## CONCLUSION

Despite global evidence supporting non-surgical scoliosis management, Canada continues to face major systemic barriers to early detection and access. Re-establishing screening practices, funding non-surgical treatment, and investing in interdisciplinary education are essential steps toward reducing the number of avoidable spinal fusions performed in this country.

## CALL TO ACTION

The Canadian Task Force on Preventative Health Care must be petitioned to revisit their 1994 archived statement that cites insufficient evidence to recommend for or against scoliosis screening and at the very least advocate for the Grieg Health Record to reconsider its recommendation against screening. It is certainly appropriate to restart the national discussion on scoliosis screening, starting with its role in routine, preventative care with an aim to prioritize earlier detection of curvatures amenable to conservative management.

## AUTHORS BIOGRAPHY

**Andrea Lebel** is a Registered Physiotherapist in Ontario with over 40 years of clinical experience and specialized expertise in the conservative management of scoliosis and spinal deformities. She opened the first Schroth clinic in Canada and was appointed as a Schroth teacher by Christa Lehnert-Schroth, playing a foundational role in advancing scoliosis-specific physiotherapy in Canada. She is the Co-Founder of Scoliosis and Spine Online Learning (SSOL), one of the world's largest scoliosis education platforms. Andrea is an SSOL international course educator in both the Schroth and Lyon Methods. She is a founding member of the Canadian Scoliosis Screening Coalition, the founder of the Scoliosis Resource Centre and the founder of the Annual Scoliosis Awareness Walk Ottawa, a CHEO fundraising event since 2012. An international lecturer, Andrea has taught certification courses and presented across four continents. She is a past SOSORT Board Member and former President-Elect, advancing evidence-based scoliosis rehabilitation worldwide.

**Miaya Kline** holds a Bachelor of Arts degree in Psychology with a minor in Health Sciences from the University of Ottawa and is currently pursuing advanced studies at the Canadian Academy of Osteopathy. Kline serves as the Media Specialist for the Canadian Scoliosis Screening Coalition and is a Lead Organizer at the Scoliosis Resource Centre. Drawing from her lived experience with Adolescent Idiopathic Scoliosis (AIS), her advocacy work focuses on the implementation of standardized preventive scoliosis screening protocols within the Canadian healthcare system.

**Josée Boucher** is a physical rehabilitation technologist at the regional pain clinic of the CISSS de l'Abitibi-Témiscamingue (Rouyn-Noranda) since 1998. She holds a college diploma in Physical Rehabilitation Techniques (Collège Montmorency) and a graduate microprogram in Strategic Intervention (UQAT). With over 25 years of clinical experience, she treats complex chronic pain conditions in adults and children, including complex regional pain syndrome (CRPS), fibromyalgia, persistent low back and neck pain, chronic abdominal pain, migraines, and mixed headaches. She contributed to the development of the Imaging Rehabilitation Program (PRI) and is involved in Interactional Pain Schools since 2008. She teached at UQAT for 11 years and helped design an education program for healthcare professionals on chronic pain, CRPS, and strategic intervention. Certified in Schroth SSOL (Practices 1 and 2), she specializes in physiotherapeutic scoliosis-specific exercises (PSSE) and postural rehabilitation of the spine. She is coauthor of scientific publications and regularly gives training and lectures throughout Quebec.

**Jacob Carberry** is a fifth-year medical student at Palacký University in the Czech Republic with a strong interest in scoliosis and spinal deformity care. Diagnosed in adolescence with a significant spinal curvature and initially offered surgery at age 13, he avoided operative treatment through bracing and intensive Schroth method physiotherapy throughout high school and early adulthood. This personal experience has shaped his commitment to early detection, conservative management, and patient-centered care. Jacob has been a long-standing volunteer with the Annual Scoliosis Awareness Walk in Ottawa, a fundraising event for CHEO, and is an active member of the Canadian Scoliosis Screening Coalition. As a future physician, he is particularly interested in the role of early screening and timely non-surgical intervention in improving patient outcomes, quality of life, and reducing the need for invasive treatment.

**Nadina Adulovic** is a Biomedical Science student at the University of Ottawa with a strong interest in scientific research and evidence based healthcare. Her academic work focuses on the application of biomedical research to improve clinical understanding and inform professional practice. She has a particular interest in research related to spinal conditions, including scoliosis, and is interested in contributing to interdisciplinary research and scholarly publications that support innovation, critical analysis, and advancements in healthcare.

**Jennifer Anne Dermott** is a Physical Therapy Practitioner at the Hospital for Sick Children, specializing in spine care and conservative management of scoliosis. With a PhD in Medical Science from the University of Toronto, Jennifer's research focuses on late presentation of adolescent idiopathic scoliosis and optimizing timely care. She has received awards for clinical teaching and research, and serves as a co-investigator on major grants advancing scoliosis diagnostics and treatment. Jennifer is widely published in peer-reviewed journals and regularly presents at national and international conferences. Her clinical expertise includes radiographic evaluation, spinal casting, and program development, while her academic roles span adjunct lecturing and committee appointments. Jennifer is committed to improving musculoskeletal health outcomes for children through evidence-based practice, research, and education.
